# Differential Effects of Calcium Alginate and Carboxymethylcellulose Wound Dressing Extracts on Human Sensory Neuron Regeneration and Secretome

**DOI:** 10.1111/wrr.70182

**Published:** 2026-06-17

**Authors:** Maximin Serbier, Gilles Carpentier, Maria Sbeih, Laura Duciel, Céline Des Courtils, Alexis Desmoulière, Amandine Rovini

**Affiliations:** ^1^ UR 20218 NeurIT, Faculties of Medicine and Pharmacy University of Limoges Limoges France; ^2^ UR 4397 Gly‐CRRET University of Paris‐Est Créteil Créteil France; ^3^ Laboratoires Brothier Nanterre France

**Keywords:** nerve ending repair, wound dressing, wound healing

## Abstract

The peripheral nervous system is critical for wound healing, with sensory nerves releasing neuropeptides that drive tissue repair. Sensory nerve damage impairs healing and contributes to chronic wounds, as observed in diabetic neuropathy. However, the impact of advanced wound dressings on peripheral nerves remains unexplored. Identifying dressings that favour nerve regeneration would aid in managing complex wounds. This study explores the in vitro effects of three wound dressings on sensory neurons: a pure calcium alginate dressing (Alginate), a mixed calcium alginate combined with carboxymethylcellulose dressing and a pure carboxymethylcellulose (CMC) dressing. Sensory neurons from neural progenitors were exposed to dressing extracts for 5 days. Cell viability, neurite regeneration after axotomy and neurite outgrowth from neurospheres were assessed. Neurite extension was evaluated in compartmentalised chips where extracts were restricted to the neurite compartment. Only Alginate was non‐cytotoxic across tested concentrations. In the axotomy model, Alginate and CMC preserved neurite regeneration, whereas the mixed dressing impaired regeneration. In neurospheres, Alginate significantly enhanced neurite outgrowth, while CMC had no effect and the mixed dressing inhibited outgrowth. Additionally, Alginate upregulated the secretion of neurotrophic and pro‐repair factors, including brain‐derived neurotrophic factor and vascular endothelial growth factor, whereas CMC and the mixed dressing mostly down‐regulated key factors. Finally, in compartmentalised culture, Alginate tended to enhance neurite extension, while CMC preserved it and the mixed dressing led to regression. These findings suggest that pure calcium alginate dressing supports nerve repair, essential for healing neuropathic wounds and may explain its good clinical performance in complex wounds.

## 1 Introduction

Chronic wounds represent a major clinical and socioeconomic challenge worldwide, affecting 1 to 2% of the population in developed countries [1]. A wide range of advanced wound dressings have now been developed with the aim of improving their treatment.

Many of these dressings have been evaluated on cutaneous cells, including immune cells, endothelial cells, fibroblasts and keratinocytes [2, 3, 4, 5, 6, 7, 8, 9, 10]. However, their impact on the peripheral nervous system (PNS) remains largely overlooked despite its well‐known role in the wound microenvironment and healing processes. Sensory nerve endings in the skin secrete neuropeptides that act on specific receptors expressed by key wound‐healing cells such as endothelial cells, immune cells, fibroblasts and keratinocytes. These interactions modulate immune function, regulate cytokine secretion, angiogenesis, collagen remodelling, cell migration and proliferation and thus drive effective wound healing [11, 12, 13, 14].

In addition, in vivo denervation studies have shown that nerve damage severely impairs inflammation, angiogenesis, collagen deposition and re‐epithelialization and leads to delayed wound healing and even to tissue necrosis [15, 16, 17, 18, 19, 20]. Clinically, this is reflected in the elevated rates of foot ulceration among patients with hereditary sensory neuropathies and in the increased risk of foot ulcers in diabetic patients [12, 13, 21, 22, 23, 24]. Diabetes itself ranks as one of the leading causes of chronic wounds [25], with over 500 M adults (20–79 years) affected worldwide; a number that is estimated to increase by 45% by 2050 [26].

Beyond the passive protective role of modern wound dressings, recent advances in wound dressings have highlighted the importance of scaffold composition and bioactive signalling in regulating tissue regeneration processes [27, 28]. In particular, alginate‐based dressings are widely used due to their high absorbency and release of bioactive Ca^2+^ ions [8, 9, 10, 27, 29]. Given the central role of sensory nerves in orchestrating wound repair, it is therefore plausible that wound dressings may also directly influence neuronal survival, neurite regeneration and neurosecretory activity.

Despite these insights, our systematic review of the literature over the last 10 years identified no studies evaluating the effects of advanced wound dressings on the peripheral nervous system. This important knowledge gap led us to investigate, in vitro, the effects of wound dressing extracts on human sensory neurons, with a particular focus on neuronal viability, neurite regeneration and neurosecretory responses associated with tissue repair. To comprehensively evaluate neuronal responses, we combined complementary in vitro models addressing distinct aspects of peripheral nerve repair. These included an axotomy model to investigate neurite regeneration following injury, a neurosphere model to assess neurite outgrowth and network organisation in a three‐dimensional environment and a compartmentalised microfluidic model mimicking the clinical situation in which wound dressings primarily interact with distal nerve endings rather than neuronal cell bodies. We selected three non‐occlusive dressings that are commonly used for both acute and chronic wounds, such as diabetic foot ulcers: a pure calcium alginate (Algostéril), a calcium alginate mixed with carboxymethylcellulose—CMC (Biatain Alginate) and a pure hydrofiber CMC (Aquacel Extra).

## 2 Materials and Methods

### 2.1 Cell Culture

**FIGURE 1:**
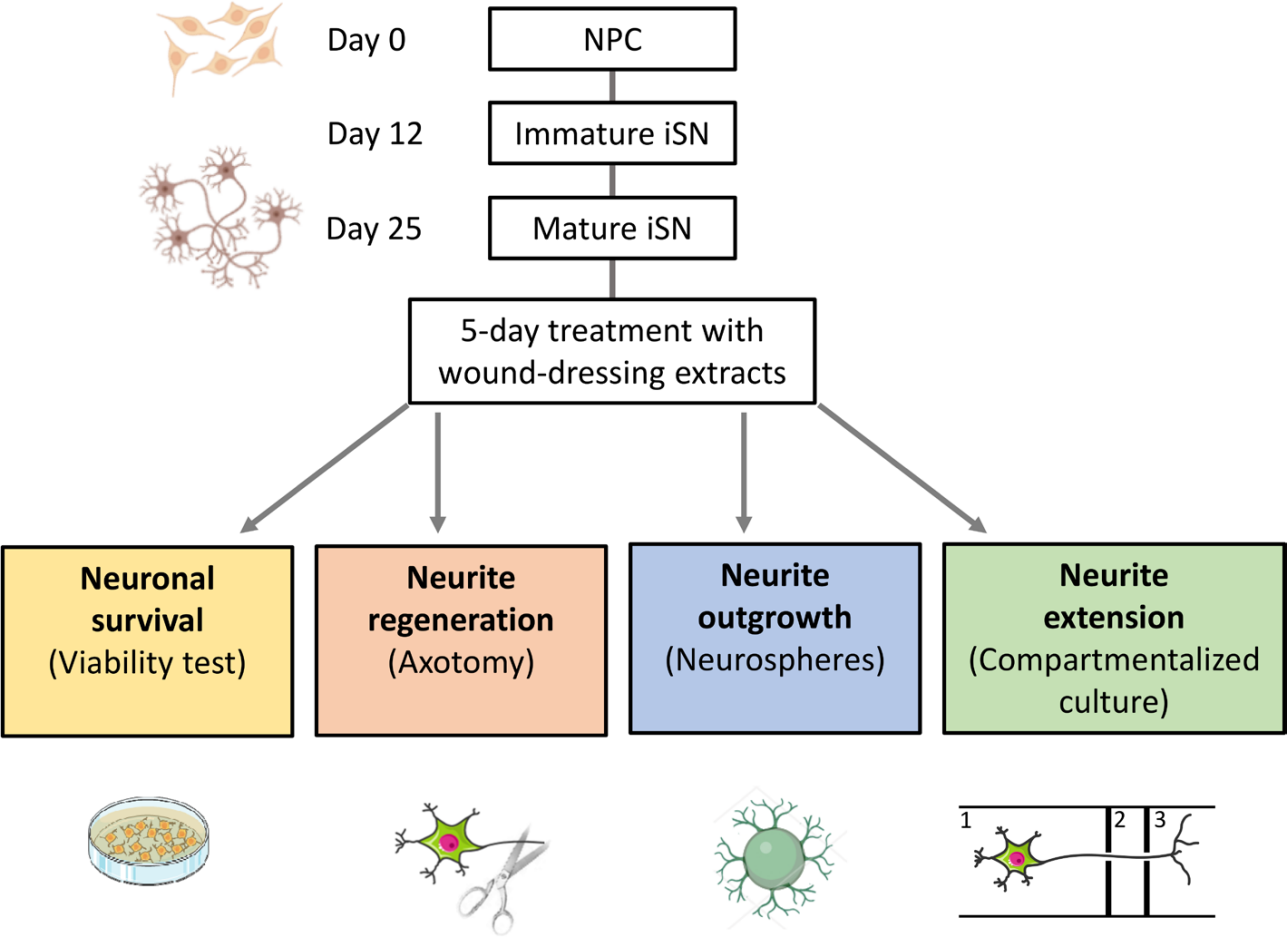
In vitro workflow for evaluating effects of wound‐dressing extracts on human induced sensory neurons. iSN, induced‐sensory neurons; NPC, neural progenitor cells.

Human induced‐pluripotent stem cell (iPSC)‐derived neural progenitor cells (NPCs) (PCi‐NPC_PC069, Phenocell, Grasse, France) were cultured on Geltrex‐coated plates in Neural Differentiation Medium (NDM). At 80% confluence, cells were re‐plated for differentiation into induced sensory neurons (iSN); when neural rosettes were formed, differentiation was induced by treatment with 10 μM DAPT (Selleckchem, Houston, Texas) and 3 μM CHIR99021 (Sigma‐Aldrich, St. Louis, Missouri) for 3 days [30]. When cells began to cluster and display short extensions, they were then cultured on appropriate culture plates for subsequent experiments. Immature iSN were maintained for 3 days in NDM supplemented (NDM+) with neurotrophic factors (Peprotech, Cranbury, New Jersey), ascorbic acid (Sigma‐Aldrich, St. Louis, Missouri) and cyclic AMP (Sigma‐Aldrich, St. Louis, Missouri), then cultured for 7 days in NDM+ with reduced supplement concentrations to obtain mature iSN. Mature iSN were characterised as previously described [31]. This work with human iPSC‐derived sensory neurons has shown progressive neurite outgrowth and morphological network maturation over multi‐day culture periods relative to initial plating and that meaningful changes in neurite networks are best quantified after several days rather than short acute exposures (e.g., significant increase in neurite length after 5 days in vitro). Figure 1 provides a workflow diagram summarising the experimental design and assays performed.

### 2.2 Wound Dressings

Algostéril (Laboratoires Brothier, Nanterre, France), Biatain Alginate (Coloplast, Humlebæk, Denmark) and Aquacel Extra (ConvaTec, London, UK) were selected for this study. Algostéril is a pure calcium alginate dressing, Biatain Alginate is composed of calcium alginate combined with carboxymethylcellulose (CMC) and Aquacel Extra is a hydrofiber dressing composed of CMC. These non‐occlusive dressings are indicated for moderately to heavily exudative wounds, both acute and chronic.

### 2.3 Preparation of Dressing Extracts

Dressing extracts were prepared according to ISO 10993‐12:2012 standard. Briefly, dressings were cut into equal‐size squares and weighed. The volume of NDM+ medium was adjusted based on the pre‐determined absorption capacity of each dressing to achieve an extraction ratio of 100 mg/mL. Samples were incubated for 24 h at 37°C under agitation (50 rpm). Obtained extracts were subsequently filtered (0.45 μm) and then diluted in culture medium. Concentrations of 30% and 50% were tested to evaluate neuronal viability. Based on viability assay results, 30% extracts were used for all subsequent neurite and secretome analyses.

### 2.4 Cell Viability Assay

Immature iSN were seeded at 45,000 cells/cm^2^ and cultured for 10 days in NDM+ medium until maturation. Cells were then incubated in NDM+ alone (control) or the dressing extract for 5 days, with medium renewed on day 3. On day 5, cells were incubated with 2 μM calcein AM (Thermo Fisher Scientific, Waltham, Massachusetts) for 20 min at 37°C. Calcein fluorescent signal, indicative of viable cells, was measured using a Fluoroskan FL microplate reader (Thermo Fisher Scientific). Experiments were performed four times.

### 2.5 Calcium Quantification in Extracts

Calcium concentrations in dressing extracts were quantified at the Department of Biochemistry and Molecular Biology, University Hospital Dupuytren (Limoges, France). Measurements were performed using a Cobas Roche C702 automated analyser (Roche Diagnostics) based on the 5‐nitro‐5′‐methyl BAPTA colorimetric method. This assay relies on the calcium‐dependent binding of Ca^2+^ to the chromogenic chelator 5‐nitro‐5′‐methyl BAPTA, resulting in a change in absorbance proportional to calcium concentration. Extracts prepared as described above were analysed directly without further dilution. Calcium concentrations were determined in five independent measurements (*n* = 5) and expressed in millimolar (mM).

### 2.6 Neurite Regeneration Assay After Axotomy

This model was designed to mimic neurite injury and evaluate regenerative capacity following neuronal damage. Mature iSN were subjected to chemical and mechanical axonal injury. Media was then removed and replaced with Accutase detachment solution (Stemcell Technologies, Vancouver, Canada) for 30 min. Cells were then washed and gently pipetted to detach the cell bodies and re‐plated on dry poly‐D‐lysine and laminin‐coated 12‐well plates as high‐density spots. Three 3 μL spots of cell suspension (33,000 cells/μL) were added per well. Spots were allowed to adhere for 10 min before carefully adding NDM+ alone (control) or medium containing dressing extracts, without detaching the spots. Medium or extracts were 50% renewed on day 3. On day 5, whole well Sartorius phase imaging was performed at 4× magnification on an Incucyte S3 Live‐Cell Analysis system (Sartorius, Göttingen, Germany). Total neurite count, junction number, covered area and length were analysed using a custom‐built plugin (G. Carpentier) as described in the phase contrast image analysis section below. Experiments were performed 5 times.

### 2.7 Neurite Outgrowth Assay From Neurospheres

**FIGURE 2:**
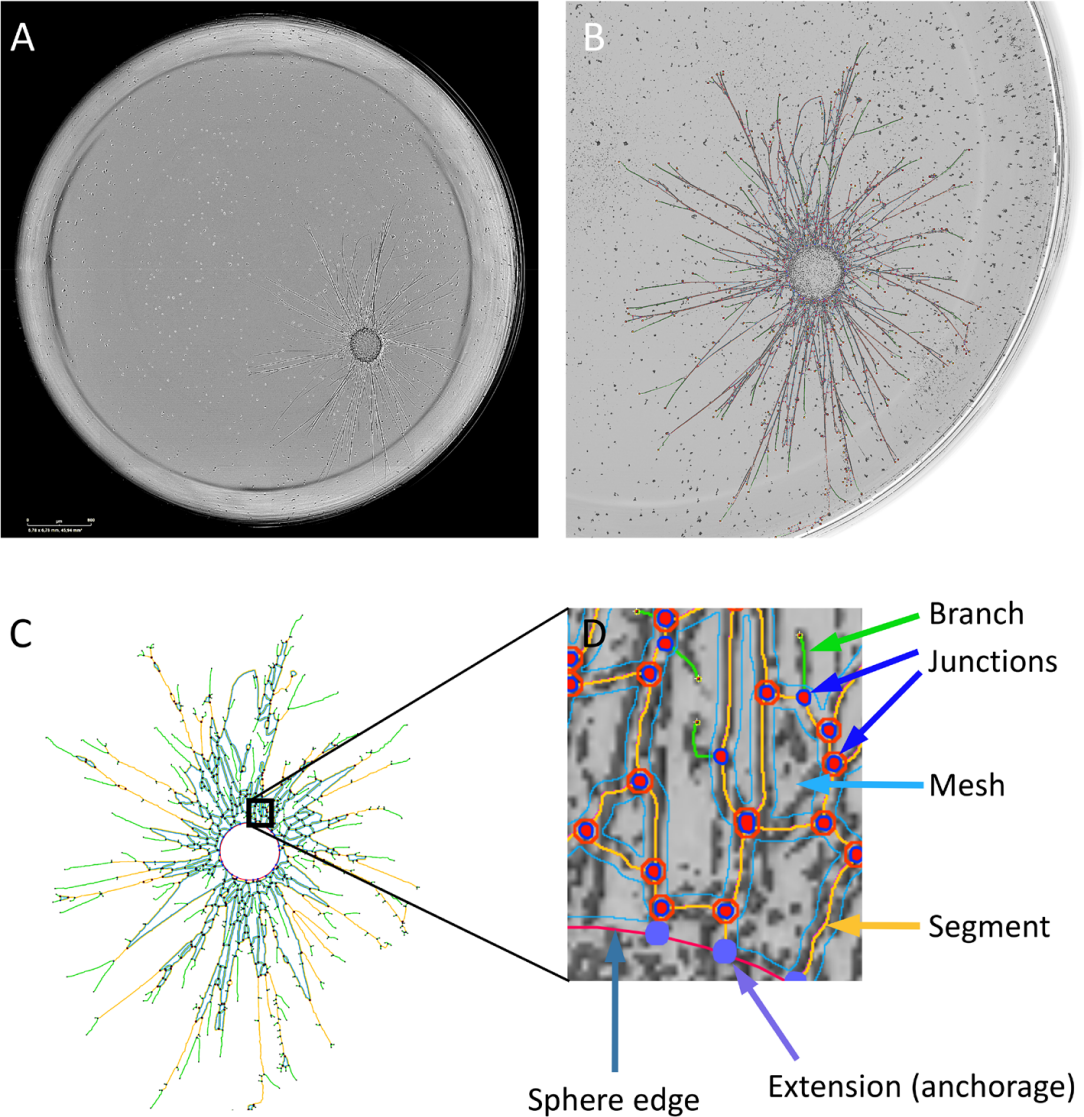
Automated morphometric workflow for neurosphere network quantification. (A) Phase contrast image of a representative well with a neurosphere. (B) Optimised detection and analysis. (C) Map of vectorial elements modelling the neurosphere structure for quantification. (D) Definition of vectorial elements: The sum of branches and segments represents the ‘total neurite length’, while the sum of mesh areas represents the ‘neurite area’.

This three‐dimensional model allowed evaluation of neurite outgrowth and neuronal network organisation while better preserving neuronal cell–cell interactions and secretory activity. Three‐dimensional sensory neurospheres were cultured as previously described [31] with slight modifications. Thirty thousand mature iSN cells were cultured in NDM+ on anti‐adherence (Anti‐Adherence Rinsing Solution, STEMCell, Vancouver, Canada) ‐treated conical 96‐well plates. After 3 days, the neurospheres obtained were individually cultured on a Geltrex‐coated 96‐well plate and then maintained for 5 days with NDM+ alone (control) or dressing extracts, with 50% medium renewal on day 3. On day 5, whole well phase imaging was performed at 4× magnification as described for the neurite regeneration assay (above). Total neurite count, junction number, covered area and length were analysed using a custom‐built plugin as described in the phase contrast image analysis section below (Figure 2). Experiments were performed five times.

### 2.8 Enzyme‐Linked Immunosorbent Assay (ELISA)

Substance P (SP) and calcitonin gene‐related peptide (CGRP) were quantified using competitive human SP (Enzo Life Sciences, Farmingdale, New York) and CGRP (Phoenix pharmaceuticals, Burlingame, California) ELISA kits according to the manufacturer's instructions. Briefly, on day 5 of neurite outgrowth assays, cells were stimulated with 1 μM capsaicin (Sigma‐Aldrich, St. Louis, Missouri) for 20 min at 37°C. Supernatants of 12 neurospheres per treatment condition were collected and incubated with a protease and phosphatase inhibitor cocktail (Sigma‐Aldrich, St. Louis, Missouri), then concentrated (×2) with an Amicon Ultra Centrifuge Filter, 3 kDa Nominal Cutoff (Sigma‐Aldrich, St. Louis, Missouri) and quantified using ELISA kits. Absorbance at 410 nm was measured with a microplate reader and SP or CGRP concentrations were calculated. Experiments were performed three times.

### 2.9 Proteome Profiler Assay

Detection of human cytokines and growth factors in response to exposure to dressing extracts was performed using the Human Cytokines Array C5 (RayBiotech, Peachtree Corners, Georgia) according to the manufacturer's instructions. Briefly, cell culture supernatants were prepared similarly to ELISA assays and incubated overnight on membrane arrays. Membranes were imaged on a ChemiDoc XRS+ system (Bio‐Rad, Hercules, California) and processed with the Protein Array Analyser ImageJ plugin. The intensity of each cytokine spot was measured based on grayscale levels and averaged across duplicate spot signals. Experiments were performed twice and targeted a selection of 80 proteins. Fold changes (FC) were calculated for each treatment condition relative to control. To ensure robustness, only proteins that were consistently up‐regulated (FC > 1) or down‐regulated (0 < FC < 1) across both experiments were considered (Table 1). Proteins with inconsistent trends and undetectable signals (FC < 0) were excluded.

**TABLE 1:** Differential regulation of selected neurotrophic, angiogenic and immunomodulatory factors in iSN neurosphere secretome following exposure to wound dressing extracts.

Functional category	Protein	Algostéril	Biatain	Aquacel
Neurotrophic	BDNF	↑	—	—
Neurotrophic	GDNF	↑↑	↑↑	↑
Neurotrophic	LIF	↑	↓	↓
Neurotrophic	NT‐4	—	↓	↓
Growth factor	IGF‐1	↑	↓	—
Growth factor	VEGF	↑	—	—
Growth factor	FGF‐9	↑	↓	—
Cytokine	M‐CSF	↑	↓	↓
Cytokine	SDF‐1	↑	—	↓
Matrix remodelling	TIMP‐1	↓	↓	↓

*Note: N* = 2; FC; Fold change; ↑, FC > 1.5; ↓, FC < 0.75; ↑↑, Fold change > 2 in both experiments.

### 2.10 Neurite Extension Assay

**FIGURE 3:**
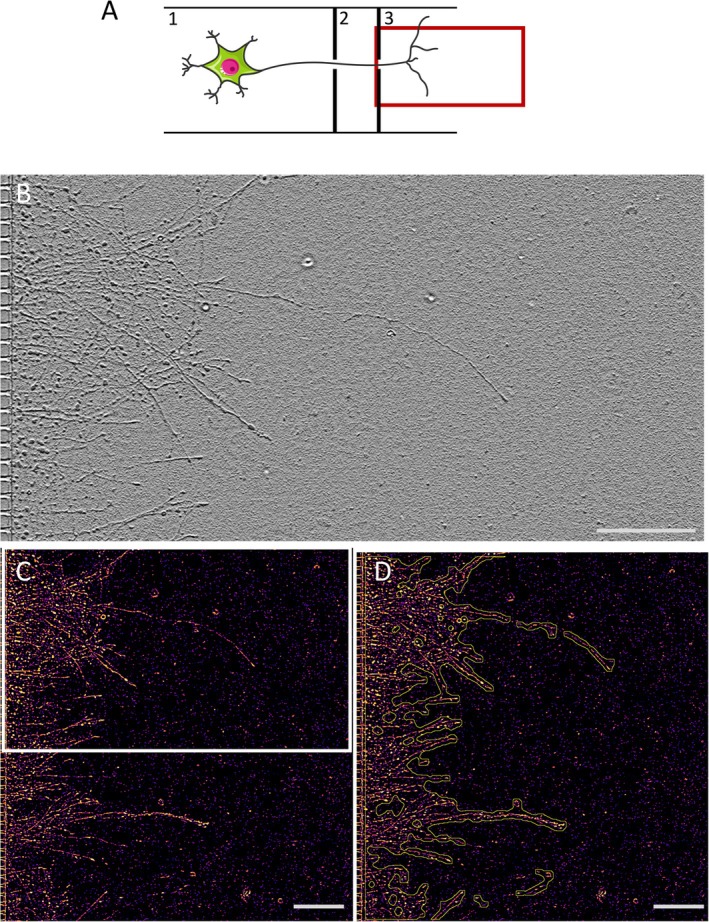
Neurite extension image analysis in NETRI DuaLink microfluidic chip. (A) Schematic of iSN culture in a NETRI chip. Extracts were exclusively added to channel 3, which contained only neurites. Red rectangle indicates the regions shown in B, C and D. (B) Zoomed‐in area of a phase contrast image after detail enhancement and background removal, scale bar 145 μm. (C) Fully optimised image prepared for binary segmentation. White rectangle indicates the region shown in B. (D) Neurite detection after segmentation, showing outlines of the quantified areas.

This compartmentalised model was used to reproduce a clinically relevant configuration in which wound dressings interact predominantly with distal neurites rather than neuronal somas. DuaLink 3‐compartment microfluidic chips (NETRI, Lyon, France) were coated with poly‐D‐lysine and laminin. Mature iSN were seeded at 30,000 cells/mm^2^ in the inlet reservoir of channel 1. Cell culture medium was replaced every 2–3 days and neurons were allowed to grow until extensions reached channel 3 (Figure 3A). NDM+ alone (control) or dressing extracts were added to channel 3 and left for 5 days, with renewal on day 3. Neurite extension was followed by phase contrast imaging on Leica DMi8 microscope (10× magnification) on day 1 and 5. Neurite progression (mm^2^) was quantified on ImageJ by manually connecting neurite tips to form a closed shape and measuring the area. Progression was then calculated as: Area (day 5)–Area (day 0). Neurite area (pixels) was quantified using a custom‐built plugin (G. Carpentier) which automatically detected neurite boundaries and calculated the precise covered area as described in the phase contrast image analysis section below (Figure 3). Area was then calculated as: Area (day 5)–Area (day 0). Experiments were performed three times.

### 2.11 Phase Contrast Image Analysis

Image analysis of phase contrast microscopy was performed with custom plugins developed for the ImageJ software.

#### 2.11.1 Neurosphere and Axotomy Models

Images were pre‐processed to reduce blur, enhance fine structures and detail and improve signal‐to‐noise ratio. Spheres were detected using specific intensity‐based thresholding methods and outlines were refined using morphological operations, center‐of‐mass evaluation, or manual adjustment (for the axotomy model). Neurite structures were then segmented using optimised masks that distinguished long peripheral extensions from densely meshed network areas. The final skeleton was obtained after removing sphere area and non‐sphere–connected objects and neurite tree structures were analysed and quantified as previously described [32] (Figure 2 represents neurosphere analysis).

#### 2.11.2 NETRI DuaLink Model

Images were processed using flat‐field correction and a series of filtering/enhancement steps to generate optimised images with enhanced structure and contrast while minimising artefacts (Figure 3B). After background correction, objects were segmented and only elongated structures were quantified. Non‐relevant areas such as chamber tracks and the soma compartment were manually excluded before measurement (Figure 3C,D).

### 2.12 Statistical Analysis

Statistical analysis was performed using GraphPad Prism. Groups were compared using the Mann–Whitney test and data was presented as mean ± Standard Error of the Mean (SEM). A *p*‐value of less than 0.05 was considered statistically significant.

### 2.13 Literature Review

A systematic review of the literature was conducted to identify studies that evaluated the effect of dressing materials on the PNS. The search strategy on Medline and Embase included English‐ or French‐language articles over the last 10 years and combined the keywords ‘Peripheral nervous system’, ‘Dressing’ and ‘Wound healing’. The search identified 86 publications, including all types of studies (in vitro, in vivo and clinical). After analysis, all of these publications were excluded for at least one of the following criteria: the study was a conference abstract, did not evaluate cutaneous tissue nor peripheral nerves, or did not evaluate dressings (but rather non‐commercial biomaterials or surgical techniques).

## 3 Results

### 3.1 Cytotoxicity of the Dressing Extracts

**FIGURE 4:**
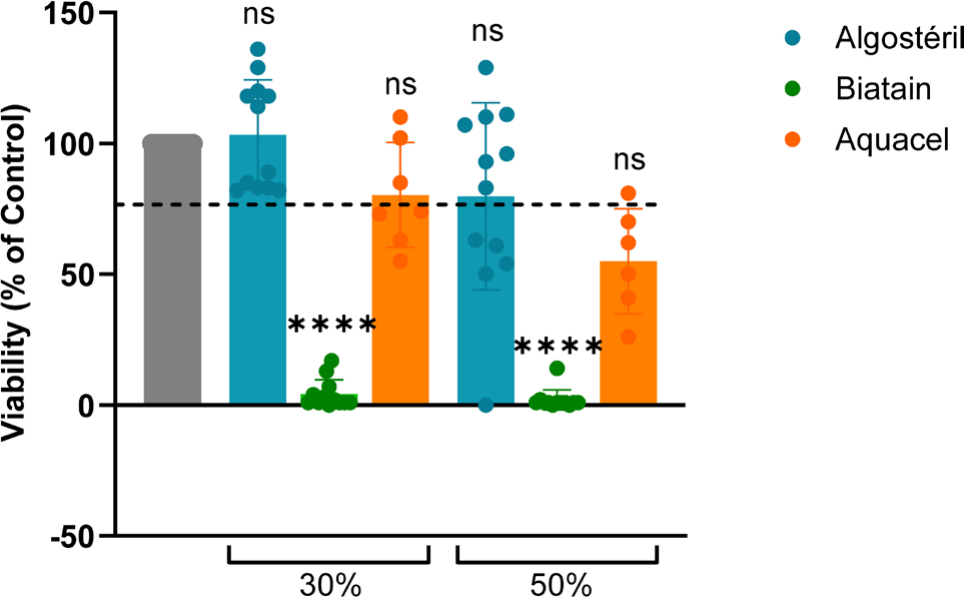
Neuronal cell viability after a 5‐day exposure to wound‐dressing extracts. Bar graph of cell viability percentage in iSN with 30% and 50% wound‐dressing extract concentrations, culture medium‐treated cells served as control (Control). Bars represent mean ± SEM of four independent experiments (*n* = 4). The horizontal dashed line (at 70% viability) indicates the cytotoxicity threshold. ****p ≤ 0.0001; ns, not significant (*p* > 0.05).

Cytotoxicity of the wound dressings (Algostéril, Biatain Alginate and Aquacel Extra) was investigated on human induced‐sensory neurons (iSNs) incubated with dressing extracts at concentrations of 30% and 50% and compared to control (culture medium alone). Viability assays revealed that only Algostéril showed no cytotoxicity (over the 70% viability threshold) at both concentrations. Aquacel Extra was cytotoxic at 50% while Biatain Alginate exhibited high cytotoxicity even at the lower concentration (Figure 4). These results identified Algostéril as the most biocompatible dressing in this model. To ensure optimal cell viability across conditions, all subsequent experiments were conducted using 30% extract concentrations.

### 3.2 Calcium Release in Extracts

**FIGURE 5:**
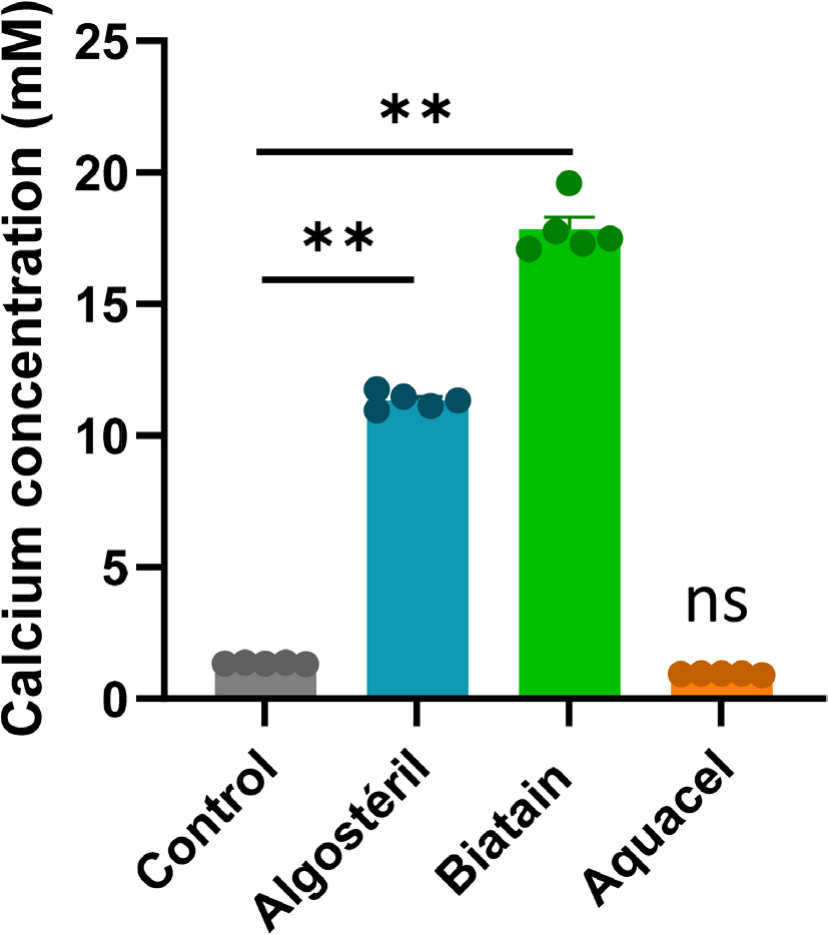
Quantification of calcium release in dressing extracts. Extracellular Ca^2+^ concentration (mM) was measured in culture medium alone (Control) or in 30% extracts of Algostéril, Biatain Alginate and Aquacel Extra after 24 h incubation. Bars represent mean ± SEM (*n* = 5). **p ≤ 0.01; ns, not significant (*p* > 0.05).

As Algostéril and Biatain Alginate both release Ca^2+^ ions, since they are composed of 100% and 85% calcium alginate respectively, we next quantified Ca^2+^ ions in 30% dressing extracts (Figure 5). Both Algostéril and Biatain Alginate increased Ca^2+^ levels with concentrations of 11.34 mM and 17.85 mM respectively, compared to Control (1.36 mM). In contrast, Aquacel Extra extract contained 0.95 mM Ca^2+^. These findings confirm substantial differences in calcium availability between alginate‐containing dressings under the experimental conditions used.

### 3.3 Effect of Dressing Extracts on Neurite Regeneration

**FIGURE 6:**
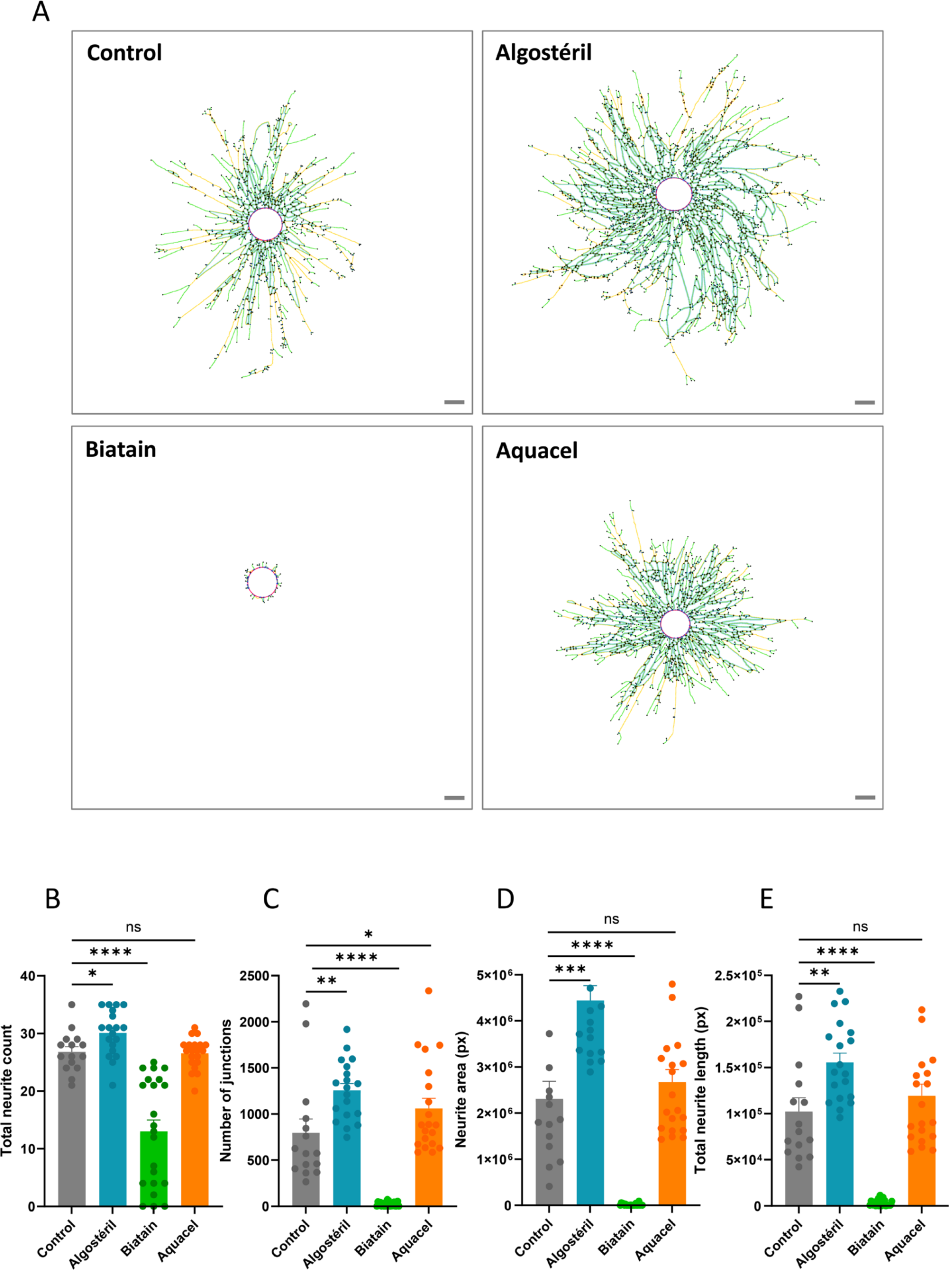
Effect of wound‐dressing extracts on neurite regeneration in axotomy model. (A) Representative images of iSN after axotomy and 5‐day treatment with 30% (v/v) Algostéril, Biatain Alginate, or Aquacel Extra wound‐dressing extracts; culture medium‐treated iSN served as control (Control). Scale bars, 500 μm. (B–E) Automated morphometric analysis of five independent experiments (*n* = 5; individual data points shown). Bars represent mean ± SEM. (B) Total number of neurites emerging from the cell body. (C) Total number of junctions (branch points) within the network. (D) Total area covered by the neurite network. (E) Cumulative neurite length. **p ≤ 0.01; ns, not significant (*p* > 0.05).

To investigate the impact of wound dressings on neurite regeneration, neurite damage was induced using an axotomy model simulating injury, followed by treatment with dressing extracts. Neurite regeneration was analysed on day 5 (Figure 6A) by quantifying total neurite count, junction number, area and length (Figure 6B–E). Algostéril and Aquacel Extra did not affect neurite regeneration as compared to control medium while Biatain Alginate significantly inhibited all four parameters. These results indicate that Algostéril and Aquacel Extra preserved neurite regeneration while Biatain Alginate had a deleterious effect.

### 3.4 Effect of Dressing Extracts on Neurite Outgrowth

**FIGURE 7:**
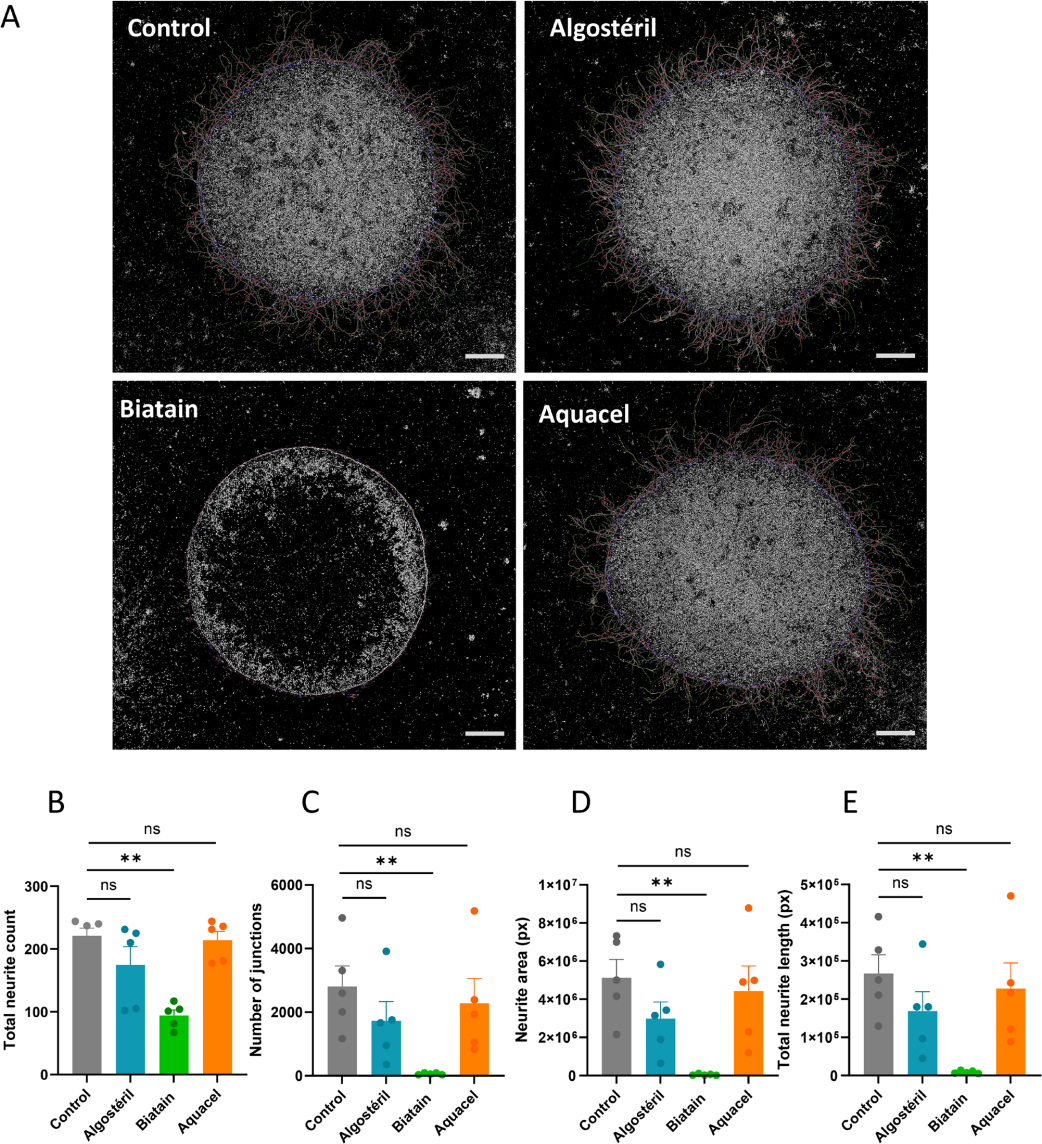
Effect of wound‐dressing extracts on neurite outgrowth in iSN neurospheres. (A) Skeletonized (vectorial) renderings of neuritic outgrowth from iSN neurospheres after 5‐day treatment with 30% (v/v) Algostéril, Biatain Alginate, or Aquacel Extra wound‐dressing extracts; culture medium‐treated cultures served as control (Control). Scale bars, 200 μm. (B–E) Automated morphometric analysis of five independent experiments (*n* = 5; individual data points shown). Bars represent mean ± SEM. (B) Total number of neurites emerging from the spheroid edge. (C) Total number of junctions (branch points) within the network. (D) Total area occupied by the neurite network. (E) Cumulative neurite length. *p ≤ 0.05, **p ≤ 0.01, ***p ≤ 0.001, ****p ≤ 0.0001; ns, not significant (*p* > 0.05).

To investigate the impact of wound dressings on neurite formation, iSN 3D neurospheres (NS) were treated with the extracts. Neurite outgrowth was analysed on day 5 (Figure 7A) by quantifying total neurite count, junction number, area and length (Figure 7B–E). Only Algostéril promoted neurite outgrowth in NS, with total count, length, covered area (reflecting density) and junction number (reflecting network complexity) all significantly increased compared to control. In contrast, Aquacel extra showed no significant influence when compared to control and Biatain Alginate showed a strong inhibitory effect. These findings point to distinct effects of each dressing on neurite outgrowth.

### 3.5 Effect of Dressing Extracts on Neuropeptide, Cytokine and Growth Factor Secretion

As neurite extension and more generally wound healing, are modulated by neuronal secretion of neuropeptides, cytokines and growth factors, we then quantified soluble factors released by NS after exposure to dressing extracts, in comparison to untreated (control) NS.

**FIGURE 8:**
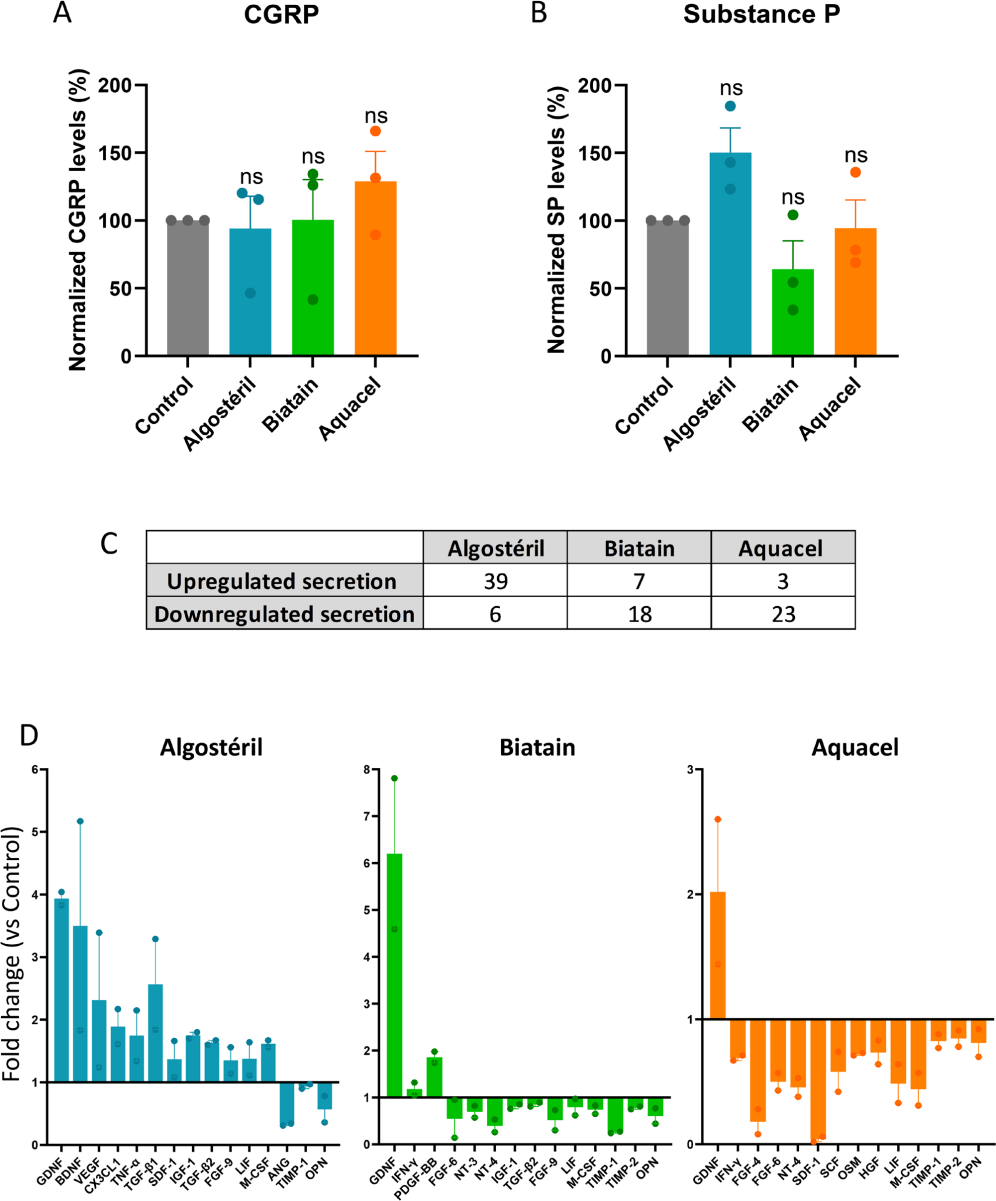
Effect of wound‐dressing extracts on neuropeptide, cytokine and growth factor release by iSN neurospheres. (A, B) Neuropeptide release measured by ELISA: (A) Calcitonin gene‐related peptide (CGRP) and (B) Substance P. Normalised levels (%) are shown from three independent experiments (*n* = 3). Bars represent mean ± SEM. ns, not significant. (C) Number of upregulated and downregulated secreted factors with each wound‐dressing extract, detected with two independent experiments (*n* = 2) of Proteome Profiler Human Cytokine Array (80 targets). (D) Fold‐change profiles of a representative subset of trophic and immunomodulatory factors. Bars represent mean ± SEM. Horizontal black line = no change vs control (fold‐change = 1).

The neuropeptide calcitonin gene‐related peptide (CGRP) levels were similar across all conditions (Figure 8A), with only a slight (non‐significant) increase observed with Aquacel Extra. For Substance P (SP), secreted levels showed a slight, non‐significant increase with Algostéril and a decrease with Biatain Alginate. Aquacel Extra showed comparable levels to control (Figure 8B).

The proteome profiler array assessed the levels of 80 secreted factors. Algostéril treatment resulted in upregulation of 39 factors and downregulation of 6. Biatain Alginate treatment resulted in upregulation of seven factors and downregulation of 18, while Aquacel Extra treatment resulted in upregulation of three factors and downregulation of 23 (Figure 8C; Table 1). All three dressing extracts increased secretion of glial cell line‐derived neurotrophic factor (GDNF) with average fold changes of 4, 6 and 2 for Algostéril, Biatain Alginate and Aquacel Extra, respectively. Algostéril alone upregulated the secretion of neurotrophin brain‐derived neurotrophic factor (BDNF) and neurotrophic leukaemia inhibitory factor (LIF). Additionally, factors including fibroblast growth factor (FGF)‐9, transforming growth factor (TGF)‐β1 and ‐β2, insulin‐like growth factor (IGF)‐1 and vascular endothelial growth factor (VEGF), all associated with key neurotrophic and wound healing roles, were all upregulated by Algostéril. Algostéril also increased the secretion of immunomodulatory cytokines such as macrophage colony‐stimulating factor (M‐CSF), stromal cell‐derived factor (SDF)‐1 and tumour necrosis factor (TNF)‐α. In contrast, Biatain Alginate downregulated LIF, FGF‐9, FGF‐6, TGF‐β2, M‐CSF and Neurotrophin (NT)‐3 and NT‐4. Similarly, Aquacel Extra induced downregulation of LIF, FGF‐4, FGF‐6, M‐CSF, SDF‐1, NT‐4 and the neurotrophic factor Oncostatin M (OSM) (Figure 8D). Together, these findings indicate that only Algostéril promoted a pro‐regenerative secretome enriched in neurotrophic and wound healing mediators, potentially explaining its ability to enhance neurite outgrowth.

### 3.6 Effect of Dressing Extracts on Neurite Extension in Compartmentalised Culture

**FIGURE 9:**
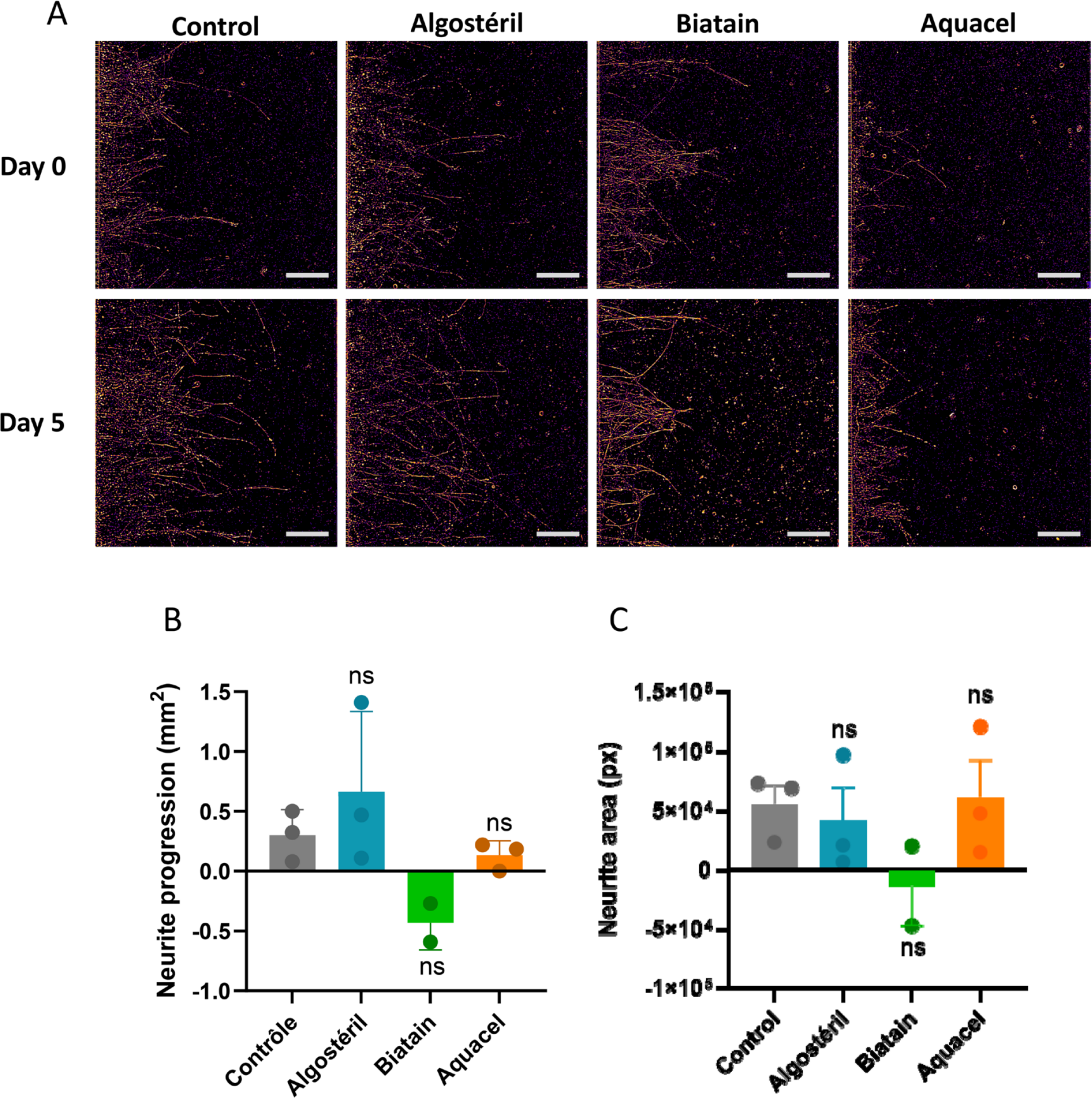
Effect of wound‐dressing extracts on neurite extension in compartmentalised culture. (A) Representative vectorized images of neurites growing through microfluidic micro‐grooves in channel 3 of the NETRI DuaLink chip at Day 0 and Day 5 after treatment with 30% (v/v) Algostéril, Biatain Alginate, or Aquacel Extra wound‐dressing extracts; culture medium‐treated iSN served as control (Control). Scale bars, 100 μm. (B) Neurite progression (mm^2^), reflecting spatial spreading of neurite tips between Day 0 and Day 5. (C) Neurite area (pixels), reflecting net neurite surface coverage between Day 0 and Day 5. Bars represent mean ± SEM of three independent experiments (*n* = 3). ns, not significant.

Since dressings are usually in contact with nerve endings rather than cell bodies, the impact of dressings on neurite extension was assessed in a compartmentalised culture where dressing extracts were added only to the neurite compartment. Neurite progression (Figure 9A,B), defined as the spatial spreading of neurite tips and neurite area (Figure 9A,C), reflecting precise surface coverage, were quantified on day 5 relative to day 0 of treatment. Algostéril showed a non‐significant trend towards enhanced neurite progression. Aquacel Extra was comparable to control while Biatain Alginate induced regression of neurites (Figure 9B). Neurite area with Algostéril and Aquacel Extra was similar to control while Biatain Alginate induced regression (Figure 9C).

## 4 Discussion

Chronic wounds represent a major global healthcare challenge, particularly in view of the increasing global prevalence of diabetes, one of the leading causes of impaired healing [25]. Many advanced wound dressings, used for treatment of chronic wounds, have previously been evaluated in contact with key wound‐healing cells [2, 3, 4, 5, 6, 7, 8, 9, 10]. However, no study in the past decade has evaluated advanced dressings in contact with peripheral nerves, despite the crucial role of the PNS in wound healing. Therefore, the results of this study are of particular interest. Three non‐occlusive dressings were evaluated in contact with human induced sensory neurons (iSN): a pure calcium alginate (Algostéril), a calcium alginate mixed with CMC (Biatain Alginate) and a pure hydrofiber CMC (Aquacel Extra).

Viability assays, conducted with dressing extracts at 30% and 50% concentrations in 2D cultures, showed that only Algostéril was non‐cytotoxic at both concentrations, while Biatain Alginate showed high cytotoxicity and Aquacel Extra moderate cytotoxicity. When tested undiluted (single replicate, data not shown), Algostéril was still non‐cytotoxic while Biatain Alginate and Aquacel Extra greatly reduced cell viability. The difference in cell viability between the two calcium alginate dressings, Algostéril and Biatain Alginate, aligns with previous results obtained on keratinocytes [2], fibroblasts [9, 29] and monocytes [10]. The cytotoxicity of Biatain Alginate may be due to toxic contaminants (trace elements) which either come from insufficiently purified alginate extracted from seaweed as described in literature [33, 34, 35, 36] or from the manufacturing‐related residues introduced during process [37]. Preliminary mass spectrometry analyses did not detect high concentrations of a compound that could explain this cytotoxicity. It may rather result from the combined presence and unknown interactions of multiple contaminants. Concerning Aquacel Extra, reduced cell viability has been observed even in keratinocytes, a less fragile cell model [2].

Because peripheral nerve repair involves multiple biological processes including neurite regeneration after injury, neurite outgrowth and distal neurite extension, dressing extracts (30% concentration) were tested on three different cell culture models: axotomy (neurite regeneration), neurosphere (neurite outgrowth) and compartmentalised (neurite extension) models.

The axotomy model was chosen to simulate, with 2D cultures, nerve damage observed in cutaneous wounds. In order to disrupt iSN neurites, chemical and mechanical means were used to detach iSN from the substrate. Following disruption, none of the dressings promoted neurite regeneration. Algostéril and Aquacel Extra performed similarly to the control, while Biatain Alginate significantly inhibited regeneration, consistent with its cytotoxicity in 2D culture.

Given the known fragility of iSN cells, exacerbated by the stress of the axotomy model, we turned to the neurosphere model which better preserves neuronal cells, enhances their neurotrophic factor secretion and mimics 3D cell–cell interactions [38]. It therefore allows better discrimination between nonspecific toxicity and modulation of neuronal signalling. In contact with neurospheres, only Algostéril enhanced neurite number, length, covered area and nodes, consistent with a maturing neuronal network. Similar to the axotomy model, Biatain Alginate and Aquacel Extra had deleterious and neutral effects, respectively. Persistence of structured neurite networks and of factor secretion (discussed later) with neurospheres in contact with Biatain Alginate suggests that the results observed cannot be attributed to generalised cell death.

Lastly, a compartmentalised microfluidic model was used to simulate the use of wound dressings in a clinical setting, as this only allows contact with neurites and not cell bodies [38]. Measurements obtained in this model indicated differences among the dressings in neurite progression and area. However, due to variability, none of these comparisons reached statistical significance. The results remained qualitatively in line with those obtained using neurospheres.

Although the present study primarily focused on neurite morphology, functional‐related neuronal responses were also investigated through neuropeptide secretion and secretome profiling. Hence, the secretome of treated neurospheres neuropeptides (SP and CGRP) were analysed by ELISA and 80 growth factors and cytokines were examined by proteome profiling. Although sensory neurons are classically associated with neuropeptide secretion, accumulating evidence indicates that peripheral neurons also produce a broad range of cytokines, chemokines and growth factors that regulate autocrine survival signalling and neuron–immune interactions during tissue repair [39, 40, 41]. The detection of neurotrophic factors (e.g., BDNF, GDNF), growth factors (e.g., VEGF, IGF‐1, FGFs) and selected cytokines in the present study is therefore consistent with neuronal secretory capacity [14].

Substance P was studied since SP interacts with neurokinin‐1 receptor (NK1R) on nerves, epithelial cells and immune cells. It enhances neuronal growth and survival by stimulating Nerve Growth Factor (NGF) secretion by keratinocytes and endothelial cells [42, 43, 44]. It also plays a major role in wound healing by recruiting macrophages, enhancing angiogenesis and modulating extracellular matrix formation [11, 12, 13, 14, 45]. Our results showed that Algostéril induced a modest increase in SP secretion which may partly explain the positive effect on neurite outgrowth observed in vitro. Biatain Alginate decreased SP while Aquacel Extra had no effect, aligning with their respective effects on neurite outgrowth. CGRP, another potent neurogenic inflammation mediator involved in neurite guidance and tissue repair [46], was secreted at a similar level across all conditions. Both SP and CGRP secretion were detectable in neurospheres in contact with Biatain Alginate, indicating some cell viability in the neurosphere model, despite the strong cytotoxicity of this dressing in 2D culture.

Proteome profiling of 80 factors revealed a predominance of upregulated factors with Algostéril (39 up vs. 6 down) while Biatain Alginate (7 up vs. 18 down) and Aquacel Extra (3 up vs. 23 down) were associated with a general downregulation of growth factors and cytokines.

Our analysis focused on 25 factors that are highly relevant for neuronal regeneration and/or wound healing (Figure 7D). Among these, neurotrophic factors GDNF, BDNF, NT‐3, NT‐4, LIF and OSM are known to support neuronal survival and differentiation as well as neurite outgrowth and extension [47, 48]. GDNF was up‐regulated across all conditions, suggesting it was not responsible for the differences observed in neurite outgrowth. These differences may instead be due to the upregulation of BDNF by Algostéril versus the down‐regulation of NT‐3, NT‐4, LIF by Biatain Alginate and the downregulation of NT‐4, LIF, OSM by Aquacel Extra.

Interestingly, Aquacel Extra and Biatain Alginate downregulated several common key factors but resulted in very different outcomes for neurite outgrowth. One possible explanation lies in their different cytotoxicity effects since high cytotoxicity can impede neurite outgrowth [49]. Another reason could be the down‐regulation of IGF‐1 observed only with Biatain Alginate. IGF‐1 has been shown to modulate and enhance the neurite‐promoting effect of GDNF in vitro [50, 51]. In fact, reduced expression of IGF‐1 in peripheral nerves has been correlated to the development of diabetic neuropathy [51]. In contrast, IGF‐1 was upregulated by Algostéril, suggesting it may be more favourable in neuropathic conditions.

Algostéril was also the only dressing that upregulated VEGF, which is involved in axonal regeneration [47, 48] as well as angiogenesis during wound healing [52]. This aligns with previous studies reporting increased VEGF secretion by fibroblasts and macrophages in contact with Algostéril, unlike the effect of Biatain Alginate [9, 10]. In contrast, the pro‐angiogenic factor angiogenin [53] was downregulated by Algostéril. These contradictory effects suggest that Algostéril may not activate all angiogenic pathways. Only two other factors, TIMP‐1 and Osteopontin, were down‐regulated in contact with Algostéril among the 25 selected. These factors that are involved in extracellular matrix remodelling [52] were also down‐regulated by contact with Biatain Alginate and Aquacel Extra, with TIMP‐2 additionally downregulated.

Finally, several FGFs, important for axonal growth, guidance and synapse formation [54] and involved in keratinocyte and fibroblast proliferation and migration during wound healing [55, 56, 57, 58], were upregulated by Algostéril (FGF‐9) and downregulated by Biatain Alginate (FGF‐6, FGF‐9) and Aquacel Extra (FGF‐4, FGF‐6).

These findings demonstrate that, of the three evaluated dressings, Algostéril is the only one that is non‐cytotoxic at all tested concentrations while also stimulating neurite outgrowth and secretion of key neurotrophic and pro‐healing factors, including BDNF, IGF‐1, VEGF and FGF‐9. Biatain Alginate and Aquacel Extra were both associated with a predominantly downregulated secretome profile under the tested conditions.

The two calcium alginate dressings, Algostéril and Biatain Alginate, produced markedly different results on sensory neurons. Both dressings release Ca^2+^ but at different levels: high for Biatain Alginate and moderate for Algostéril. This difference in Ca^2+^ release aligns with previous results and is linked to dressing composition (pure calcium alginate vs alginate combined with CMC) and to different mannuronic‐to‐guluronic acid ratios in their alginate fraction [29]. Given the known gaussian effects of extracellular calcium on cell activation, insufficient or excessive extracellular Ca^2+^ can both impair calcium signalling, a potent activator of cellular functions [59, 60, 61, 62, 63]. Excessive Ca^2+^ exposure observed with Biatain Alginate may therefore contribute to its cytotoxicity and impaired neurite responses. The superior performance of Algostéril could be attributed to the moderate concentration of released Ca^2+^, previously shown to elevate intracellular Ca^2+^ concentrations to levels optimal for triggering calcium signalling [29]. Indeed, in neurons, calcium signalling activates mechanisms responsible for neurite outgrowth [64] and secretion of neuropeptides [65]. Concerning Aquacel Extra, it is composed of linear cellulose derivatives and does not release Ca^2+^ ions, as observed in our results, which aligns with the general absence of effect on neurites.

Limitations of this study include the in vitro nature of the models used, which excludes mechanical and immune‐mediated cues of a complex wound environment. Another related limitation is the use of dressing extracts instead of complete dressings. This was done in accordance with ISO 10993–12:2012 for the evaluation of medical devices. It avoids the hypoxic effect a complete dressing would have on cultured cells and allows the evaluation of released components into the wound. Future studies should further investigate neuronal functionality using electrophysiological recordings, calcium imaging, or in vivo wound models allowing direct contact between dressings and regenerating nerve endings.

Advanced wound care research has increasingly emphasised the role of material composition, architecture and release kinetics in modulating biological responses [66]. Features such as fibre morphology, compositional ratios and incorporated functional moieties (e.g., nanoparticles) can significantly influence cellular behaviour in vitro and in vivo [27]. Consistent with this, our data suggests that alginate dressings exert complex effects on sensory neurons that likely arise from a combination of ionic release, structural cues and scaffold signalling rather than simple biocompatibility metrics alone.

In conclusion, this study demonstrates that wound dressings can differentially affect human sensory neuron survival, neurite regeneration, neurite outgrowth and neurosecretory activity in vitro. Among the three evaluated dressings, the pure calcium alginate dressing Algostéril was the only one that consistently preserved neuronal viability while promoting neurite outgrowth and the secretion of key neurotrophic and wound‐healing factors, including BDNF, IGF‐1, VEGF and FGF‐9. This may represent an additional healing mechanism, complementing the previously described activation of fibroblasts and immune cells by Algostéril [8, 9, 10]. This may further explain the clinical efficacy of Algostéril in wound healing [67, 68, 69, 70], particularly in complex wounds associated with neuropathy such as diabetic foot ulcers [71].

These findings highlight the importance of considering peripheral nerve responses when evaluating advanced wound dressings, particularly in the context of chronic and neuropathic wounds. More broadly, this work emphasises that wound dressings are not biologically inert materials and may actively influence neuro‐regenerative processes involved in tissue repair. Future studies integrating functional neuronal analyses and in vivo wound models will be important to further validate these observations and support the development of wound dressings specifically designed to promote nerve regeneration and healing.

## Funding

This work was supported by the Laboratoires Brothier, Nanterre, France.

## Conflicts of Interest

The authors declare no conflicts of interest.

## Data Availability

The data that support the findings of this study are available on request from the corresponding author. The data are not publicly available due to privacy or ethical restrictions.
